# The impact of a biocellulose-based repair of fetal open spina bifida on the need to untether the cord: is it time to unify techniques for prenatal repair?

**DOI:** 10.31744/einstein_journal/2024AO0557

**Published:** 2024-04-09

**Authors:** Denise Araújo Lapa, Gustavo Yano Callado, Giulia Catissi, Lucas Trigo, Fernanda Faig-Leite, Ana Paola Arthaud Berthet Sevilla

**Affiliations:** 1 Hospital Israelita Albert Einstein São Paulo SP Brazil Fetal Therapy Program, Hospital Israelita Albert Einstein, São Paulo, SP, Brazil.; 2 Hospital Infantil Sabara São Paulo SP Brazil Hospital Infantil Sabara, São Paulo, SP, Brazil.; 3 Hospital Israelita Albert Einstein Faculdade Israelita de Ciências da Saúde Albert Einstein São Paulo SP Brazil Faculdade Israelita de Ciências da Saúde Albert Einstein, Hospital Israelita Albert Einstein, São Paulo, SP, Brazil.; 4 Hospital Sant Joan de Déu Barcelona Cataluña España Hospital Sant Joan de Déu Barcelona, Cataluña, España.; 5 Hospital Clínic de Barcelona Fetal Medicine Research Center Barcelona Cataluña España Fetal Medicine Research Center Barcelona, Hospital Clínic de Barcelona, Cataluña, España.; 6 Hospital Samaritano Higienópolis São Paulo SP Brazil Hospital Samaritano Higienópolis, São Paulo, SP, Brazil.

**Keywords:** Spinal dysraphism, Fetoscopy, Fetus/surgery, Cellulose, Motor activity, Infant, newborn

## Abstract

**Objective:**

To report the need for cord untethering after prenatal repair of open spina bifida using a unique biocellulose-based technique performed at a later gestational age.

**Methods:**

An observational cohort study was conducted to determine the incidence of tethered cord syndrome. Between May 2013 and May 2022, we performed 172 procedures using the percutaneous fetoscopic approach in fetuses at 26-28 weeks of gestation. After placode dissection, a biocellulose patch was placed to cover the placode, a myofascial flap (when possible) was dissected, and the skin was closed. Owing to death or loss to follow-up, 23 cases were excluded. Cord tethering syndrome was defined as symptoms of medullary stretching, and the infants were evaluated and operated on by local neurosurgeons after an magnetic resonance imaging examination. Infants over 30-month had ambulation and neurodevelopment evaluations (PEDI scale).

**Results:**

Among 172 cases operated at a median gestational age of 26.7 weeks and delivered at 33.2 weeks, 149 cases were available for postnatal follow-up, and cord untethering was needed in 4.4% of cases (6/136; excluding 13 cases younger than 12 months). Cerebrospinal fluid diversion and bladder catheterization were needed in 38% and 36% of cases, respectively. Of the 78 cases evaluated at 30 months, 49% were ambulating independently, and 94% had normal social function.

**Conclusion:**

The biocellulose-based technique was associated with a low rate of cord tethering, wich may be attributed to the lack of the duramater suture during prenatal repair, the formation of a neoduramater and/or later gestational age of surgery.

## In Brief

In this study, we conducted a biocellulose-based prenatal repair of open spina bifida at later gestational ages. Of the 172 cases subjected to percutaneous fetoscopic method between 26 and 28 gestational weeks, cord untethering was needed in only 4.4% (6/136), indicating a low rate of tethered cord syndrome. Myelomeningocele correction involves the placement of a biocellulose patch and myofascial flap dissection. The results showed favorable outcomes at 30 months, with independent ambulation and social function in 49% and 94%, respectively. This study suggests that the low rate of tethering may be attributable to factors such as neoduramater formation and the absence of duramater sutures, thus providing valuable insights for future advancements in prenatal spina bifida management.

## Highlights

■ This study focused on a prenatal biocellulose-based repair technique for open spina bifida that was performed at a later gestational age (26-28 weeks).

■ Myelomeningocele repair involves placode and myofascial flap dissections, with the placement of a biocellulose patch that contributes to successful outcomes, by inducing the formation of a neodurameter.

■ Only 4.4% of patients required cord untethering, indicating a notably low rate of tethered cord syndrome associated with the procedure, especially when compared with other prenatal myelomeningocele-correction techniques.

■ The results demonstrated positive outcomes, with 49% and 94% of children achieving independent ambulation and normal social function, respectively, at 30 months.

## Graphical abstract

**Figure:**
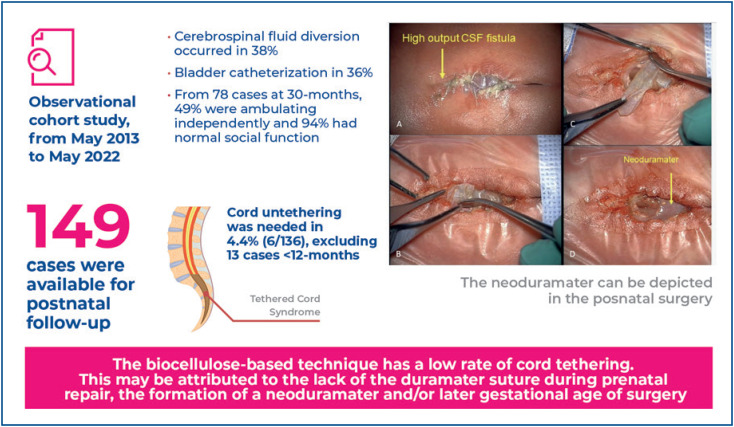

## INTRODUCTION

The Management of Myelomeningocele Study (MOMS) showed that prenatal repair of open spina bifida (OSB) mitigates motor dysfunction and the need for treatment of hydrocephalus.^(1,2)^ The Skin-over-biocellulose for Antenatal Fetoscopic Repair (SAFER), a minimally invasive percutaneous technique for innovative fetoscopic biocellulose-based repair, is aimed at improved maternal safety.^(3,4)^ Although developed for fetoscopic use, SAFER can be used in open surgery or laparotomy-assisted fetoscopic repair, and relies on the dissection and protection of the placode by using a biocellulose patch. Watertight dura closure is achieved, not with sutures, but through the formation of a biocellulose-induced neoduramater. As this neoduramater separates the spinal tissue from the muscle and skin, we posited that the neoduramater could decrease the incidence of symptomatic cord tethering (tethered cord syndrome [TCS]).

Congenital cord tethering can occur when the conus medullaris remains attached to the *filum terminalis*, or after surgical repair of spina bifida.^(5,6)^ During growth in height, TCS occurs from the stretching of the spinal cord that has adhered to the scar, and usually manifests at school age. Detected on only clinical diagnosis, TCS occurs in 20-30% of patients who have undergone neonatal repair.^(6,7)^ Symptoms of TCS include neurological dysfunction, such as changes in gait, worsening of bladder continence or intestinal function, progressive scoliosis, localized pain, impaired deglutition, and respiratory dysregulation.

The only treatment for TCS is a second neurosurgical procedure for untethering the spinal cord. Owing to the degree of adhesion of the spinal cord tissue to the scar, surgery can cause additional neurological damage and is a challenging procedure, with morbidity rates of nearly 8%, from infection/sepsis, cerebrospinal fluid (CSF) fistula, retethering of the cord, etc.^(6)^

Evaluation of the prenatal correction group of the MOMS cohort at school-age (6-7 years)^(8)^ showed a reduction in ambulation to 29%, from 42% at 30 months.^(9)^ One of the reasons for such a decrease was the significantly higher need to untether the cord in the prenatal group than in the postnatal group (27% *versus* 15%; p=0.03).^(9)^ Although the cause for this difference between prenatal and postnatal repair is largely unknown, the type of patch used and the gestational age at surgery may have some impact. As the *conus medullaris* normally ascends during pregnancy, earlier operation is associated with a lower position of the cord that can attach to the scar.^(5)^

## OBJECTIVE

We aimed to report the outcomes of prenatal biocellulose-based SAFER, with a focus on the need to untether the cord.

## METHODS

This observational cohort study is reported in conformance with the STROBE guidelines. The study was approved by the Research Ethics Committee of the *Hospital Israelita Albert Einstein* (CAAE: 48991021.0.0000.0071; #5.174.678; approved on December 17, 2021).

### Diagnosis

Ultrasonography was used to diagnose OSB associated with hindbrain herniation. Fetal magnetic resonance imaging (MRI) was performed preoperatively and postoperatively. Hindbrain herniation was defined based on the position of the cerebellar tonsils below the foramen magnum. To avoid extreme prematurity and include late referrals, surgical correction of OSB was performed from 25 to 30 weeks of gestation.

**Figure 1:**
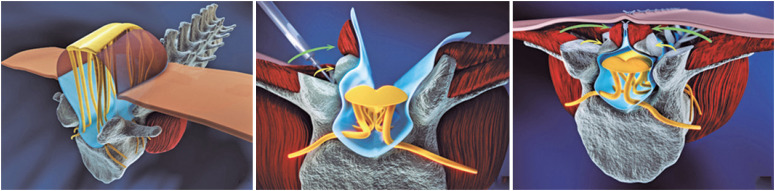
Schematic representation of the technique to dissect and rotate the myofascial flap

The technical issues associated with percutaneous fetoscopic OSB repair have been described previously.^(3,10)^ Briefly, maternal total intravenous anesthesia (TIVA) was used for both mother and fetus; in the first 34 cases, we additionally used gas (sevofluorane), which we do not use anymore (May, 2016). Ultrasound-guided placement of 3 or 4 trocars, one 5.8mm balloon-tipped (Applied Medical^®^, Rancho Santa Margarita, CA, EUA) and 2 or 3 11F vascular introducers (Terumo^®^, Tokyo, Japan). We suctioned out the amniotic fluid (and saved it if it was not bloodstained); then, we insufflated the uterus with heated and humidified carbon dioxide, usually up to 15mmHg. We then rotated the fetus into a face-down position, with the legs apart to avoid buoyancy. After maternal anesthesia, the cephalic position was maintained, and for fetuses in breech or other positions, an external rotation was performed. We released the neuroplacode from the transitional zone and placed a biocellulose patch (Bionext^®^, Bionext, São Paulo, SP, Brazil) to protect the underlying medulla. Sutures were not used to fix the biocellulose to any tissues or to primarily close the duramater. The skin was sutured at the midline with running sutures (nonabsorbable Quill™ ©2020 Surgical Specialties Corporation, Westwood, MA, USA) either directly above the patch, or above the myofascial flap whenever it was possible to dissect the flap, from case 76 onwards (the cases have been numbered consecutively since the prenatal repair surgeries were begun). Using the modified CHOP technique for open repair,^(11,12)^ the myofascial flap was dissected bilaterally, rotated toward the midline to be sutured, with care to avoid additional pressure on the placode (Figure 1). The biocellulose was fitted to cover the placode and we used running sutures (absorbable Quill©2020 Surgical Specialties Corporation, Westwood, MA, USA) to secure the flap above the patch. If the skin available was insufficient for midline closure, we used the same suture to place a skin substitute (Nevelia^®^, Symatese, Chaponost, France) to close the skin gap, thereby avoiding the need for relaxing incisions. In all cases prior to Case 78, we used mononylon 4-0 to fix another type of skin substitute (Integra© Dermal Regeneration Template, Plainsboro, NJ, USA) to the skin.

After slowly releasing the gas, the reserved amniotic fluid was returned to the uterine cavity. The trocar sites in the myometrium were not sutured; only the maternal skin was sutured.

Throughout the procedure, close monitoring of maternal expired carbon dioxide was maintained, and gas insufflation was immediately stopped to reduce the intrauterine pressure if a sudden drop was noted, and surgery was resumed when the maternal parameters had normalized.

Patients regained consciousness in the operating room and were observed for 1 hour postoperatively before being transferred to the regular maternal ward and discharged after 3 days. For patients who did not deliver in our unit, detailed instructions were provided to the obstetrician and neurosurgeon.

### Study population

Between May 2013 and May 2022, prenatal OSB repair, using the SAFER technique,^(4)^ was performed by the same surgeon in 172 consecutive patients, including 7 twin pregnancies wherein one or two affected fetuses (n=8 fetuses in total) were operated on. Among these, a total of 23 cases were excluded for the following reasons: noncompletion of repair (n=3 cases; two of these were due to loss of access to the uterine cavity because of gas leakage into the maternal abdomen, and one was due to maternal CO_2_ imbalance that necessitated immediate delivery for maternal safety); two intrauterine fetal deaths; one pregnancy termination after surgery (parental decision); and six neonatal deaths due to prematurity.

There were three additional postnatal deaths before 12 months of age, all of which were attributed to infections or complications related to ventriculoperitoneal shunts. Three deaths occurred after 12 months: one each due to ventriculoperitoneal shunt complications, complications following a second or third ventriculostomy, and a severe allergy to the contrast used during MRI, as requested by the local neurosurgeon (not part of our institutional protocol).

**Figure 2:**
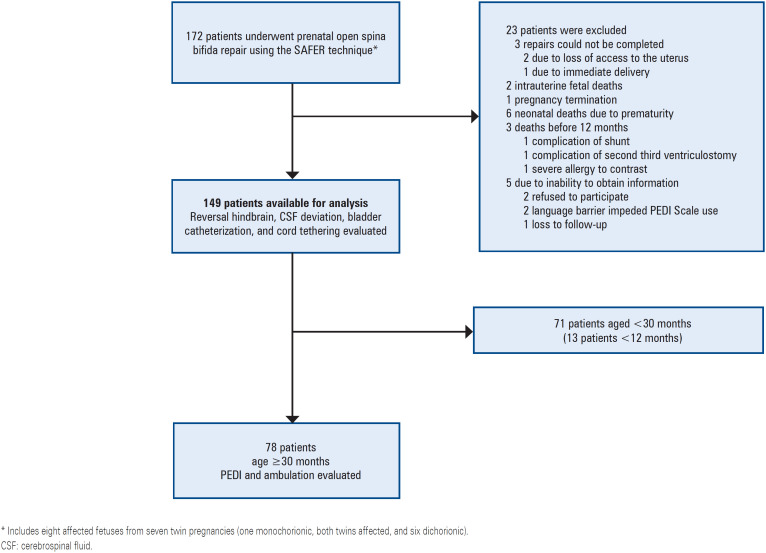
Total number of cases operated for the prenatal repair of open spina bifida, using biocellulose-based correction

We could not obtain additional information for two patients who declined to participate; two patients were affected by a language barrier that prevented the application of the Pediatric Evaluation of Disability Inventory (PEDI) Scale,^(13,14)^ and only one patient was lost to follow-up (Figure 2).

### Follow-up

**Figure 3:**
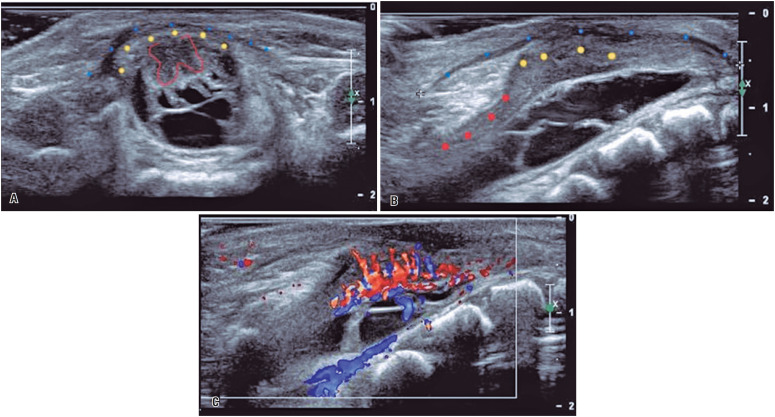
Postnatal ultrasound of the spinal column at the level of the spina bifida repair. (A) Transverse section shows the biocellulose patch (blue dots), the possible neoduramater (yellow dots), and the red line depicts the preserved anterior horns. (B) Longitudinal section showing the biocellulose patch (blue dots), the former placode (yellow dots), and the normal image of the medulla reaching the repaired area from the cranial direction (red dots). (C) At the same level, the color Doppler shows the intense vascularization of the former placode. Note: the vessels do not cross the biocellulose patch

Delivery details were obtained from a neonatal intensive care unit report, and the parents were contacted for additional information and follow-up. Postnatal characteristics, such as weight, Apgar score, and need for postnatal surgery, were recorded. When patients underwent delivery at our center, spinal ultrasound (Figure 3) was performed by a pediatric ultrasound specialist, to assess wound-healing parameters, because the use of biocellulose in fetal cases has not been reported in the literature.

Preoperative MRI was performed in all 172 patients, whereas postoperative MRI was performed in only 86 patients, either because the baby was born within 4 weeks after surgery or the parents did not agree to a second MRI. We compared the position of the cerebellum pre- and postoperatively and classified the herniation reversion as follows: "none," if the cerebellum remained in the same position; "partial," if part of the cerebellum still crossed the foramen magnum; and "complete," if the entire cerebellum was above the foramen.

At 12 and 30 months, babies routinely underwent neurological, motor, gastrointestinal, and urinary evaluations. Data that were prospectively collected, before and after surgery, and entered into the online RedCAP database. This study was approved by our Institutional Review Board (SGPP: 4667-21).

We analyzed data from our entire cohort up to April 2023 in an active search for infants with symptoms of a tethered cord. A maternal survey describing the syndrome was conducted, and cases that required surgery to untether the cord were evaluated. The operated cases were reviewed by our team; however, the decision to perform surgery was made at the discretion of the local neurosurgeon. Information regarding the need for bladder catheterization and CSF diversion was obtained from attending physicians, nurses, medical records, and caregivers. The ambulation status was assessed only in babies aged ≥30 months and, thus, 78 cases were assessed in 2022.

### The PEDI Scale

In 2022, we performed a cognitive evaluation using the PEDI Scale in infants aged ≥30 months. The PEDI Scale enables an interview-based assessment of functional capabilities and performance in three main areas: self-care, mobility, and socialization.^(13,14)^ Parents or caregivers respond to standardized questions based on their impressions and observations of the child’s development and behavior in daily life.

The PEDI Scale was chosen by our group because it has already been validated for the Brazilian Portuguese language,^(14)^ and PEDI-based interviews could be performed using telemedicine. Two PEDI-certified occupational therapists conducted the assessment. Other scales, such as Bayley’s, were not chosen because they would require the parents and children to travel to our center.

Unlike Bayley’s scale, which is limited to children aged up to 42 months, the PEDI is comprehensive and can be used for children up to 7 years of age. A systematic review conducted in 2016 showed that the PEDI was the most commonly used scale for evaluating children with disabilities in non-English-speaking populations. The PEDI comprises 197 questions, of which 73, 59, and 65 assess children’s self-care, mobility, and social functioning, respectively. When a child was considered normal in two or three of these main areas, the PEDI results were classified as normal.

## RESULTS

Of the 149 cases with data available for analysis, 78, 71, and 13 were aged ≥30, <30, and <12 months, respectively. The demographic characteristics of the 149 patients are summarized in table 1. In one case, spinal ultrasonography revealed a biocellulose patch, and a layer of new tissue was observed just below the patch (Figure 2). We believe that this layer could be a neoduramater, as it shows anatomical continuity with the original duramater.

**Table 1:** Maternal and pregnancy characteristics of patients undergoing prenatal repair of open spina bifida using the biocellulose-based repair

Variable		≥30 months (n=78)	<30 months (n=71)	Total (n=149)
Maternal age (years) at surgery		32.0 (28.1-35.0)	32.9 (29.7-36.7)	32.4 (29.2-35.7)
Maternal BMI (kg/m^2^)		26.7 (24.3-29.0)	27.1 (23.8-29.2)	26.9 (24.2-29.1)
Preconceptional folic acid use		30 (38.4)	32 (39.4)	62 (41.6)
First pregnancy		39 (50.0)	39 (54.9)	78 (52.3)
GA at surgery (weeks)		26.7 (25.6-27.4)	26.6 (25.3-27.9)	26.7 (25.4-27.5)
GA at birth (weeks)		33.1 (31.4-35.0)	33.2 (31.0-35.6)	33.2 (31.3-35.2)
	GA at birth <32 weeks	21 (26.9)	26 (36.6)	47 (31.5)
	GA at birth <30 weeks	8 (10.3)	8 (11.3)	16 (10.7)
MRI with pre- and postoperative images		49*	37*	86*
	Complete reversal HH	36 (73.5)*	28 (75.7)*	64 (74.4)*
	Any degree of reversal HH	48 (98.0)	36 (97.3)	84 (97.7)
Myofascial flap		19 (24.4)	56 (78.9)	75 (50.3)
Bladder catheterization		32 (41.0)	21 (29.6)	53 (35.6)
Age (months) at the 30-month follow-up		36.6 (30.2-39.4)	–	–
Ambulation status				
	Not walking	16 (20.5)	–	–
	Assistive device	26 (33.3)	–	–
	Independent	36 (46.2)	–	–
Shunt or ventriculostomy		36 (46.2)	21 (36.2)†	57 (38.3)†
Tethered cord surgery		4 (5.1)	2 (3.4)†	6 (4.4)
Surgery for CSF leakage		9 (11.5)	0 (0.0)	9 (6.0)
Surgery for dehiscence		9 (11.5)	4 (5.6)	13 (8.7)

Data are presented as the median (interquartile range) or the frequency (proportion [n (%)]).

* 49 cases with postoperative MRI;

† 13 cases were <12 months old, total, 136 cases.

GA: gestational age; MRI: magnetic resonance imaging; HH: hindbrain herniation; CSF: cerebrospinal fluid; BMI: body mass index.

### Outcome measures

#### Study cohort

In the entire study cohort (with 149 cases), the maternal age at surgery was 32.4 (29.2-35.7) years, median age at birth was 33.2 (31.3-35.2) weeks, and median maternal body mass index was 26.9 (24.2-29.1) kg/m^2^; moreover, 41% (62/149) of the mothers used preconceptional folic acid supplementation.

Postoperative MRI showed some degree of hindbrain herniation reversal (complete or partial) in 97.7% of cases (84/86), and complete reversal in 74.4% (64/86). Cerebrospinal fluid diversion (shunt and/or ventriculostomy) and bladder catheterization were performed in 38.3% (57/136) and 35.6% (53/149) of patients, respectively (Table 1).

### The ≥30-month sub-cohort

In the ≥30-month sub-cohort (78 cases), based on ambulation status, the children were classified into three groups: not walking 20.5% (16/78), walking with an assistive device 33.3% (26/78), and walking independently 48.7% (38/78). The mean chronological age 39.6 (29.8-95.7) months. After 30 months, 5.1% (4/78) underwent cord-untethering surgeries. The indications included dermoid cysts, progressive scoliosis, and worsening of bladder and intestinal function (Table 2).

**Table 2:** Details of cases that underwent surgery to untether the cord

Case	Case number*	Indication for surgery	Age at cord untethering (months)
1	13	Dermoid cysts	60
2	19	Progressive scoliosis	49
3	20	Worsening bladder function	26
4	26	Muscular atrophy	68
5	60	Worsening intestinal function	60
6	135	Worsening intestinal function	21

* Our cases have been numbered sequentially from the first surgery onward.

**Figure 4:**
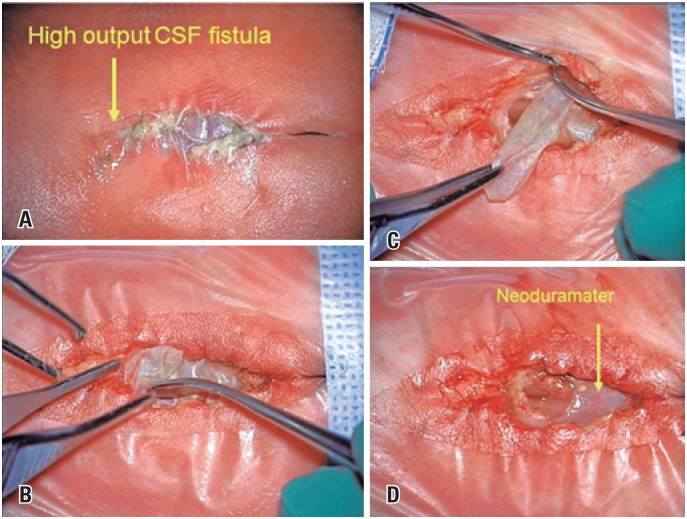
Neonate with high-output cerebrospinal fluid fistula at the repair site. (A) There is no skin dehiscence; only the biocellulose is visible; (B) and (C) Images of the posnatal surgery to repair a high-output cerebrospinal fluid fistula observed at birth in one of our cases. (D) Note the oval aspect of the neoduramater defect from where the fluid is leaking. The preserved placode is in the center of the lesion. Repair was uneventfull as the biocellulose does not adhere to the surrounding tissues

Notably, one patient was born with a high-flow CSF fistula and required postnatal neurosurgical repair. Intraoperatively, we observed the presence of biocellulose, removed the patch, and found new tissue at the duramater level that likely corresponded to neoduramater. This tissue was not completely closed at the time of birth; only a small hole that seemed responsible for the leak was primarily closed after the removal of the biocellulose patch (Figure 4).

### PEDI assessment

The PEDI assessment was performed in 76 of the 78 patients. Two patients were not evaluated using the PEDI because their caregivers could not communicate in Portuguese. A normal social function score was observed in 93.4% (71/76). A normal result was achieved in 60.5% (46/76) of the children, indicating that these children had the same abilities as children without spina bifida. In 38.2% (29/76) of patients, motor function was the only abnormal parameter on the PEDI scale. The detailed PEDI results are listed in table 3.

**Table 3:** Results of the Pediatric Evaluation Disability Inventory

Parameter		n (%)
Self-care		
	Normal	45 (59.2)
	Abnormal	31 (40.8) *
Mobility		
	Normal	17 (22.4)
	Abnormal	59 (77.6)
Social function		
	Normal	71 (93.4)
	Abnormal	5 (6.6)
Final result		
	Normal	46 (60.5)
	Abnormal	30 (39.5)

* Parents frequently performed tasks with helping the children.

### The <30-months group

Of the 71 patients aged <30 months at follow-up, 58 were between 12 and 30 months old, and 13 were less than 12 months old. Two patients (3.4%; 2/58), required cord untethering - one case at 21 months and the other at 26 months due to worsening of bladder and intestinal function, respectively; these systemic functions improved after surgery (Table 2).

## DISCUSSION

The PEDI scale-based evaluation of neurodevelopment showed that >90% of the babies had normal social function, and >60% achieved a normal final result. This indicates that these infants were capable of performing daily activities similar to other children of the same age without disabilities.

Of the total of 78 cases aged ≥30 months, 58 were aged 12 to 30 months, the need to untether the cord was 3% and 5%, respectively. These numbers are lower than those for any other prenatal repair techniques reported in the literature. However, statistically significant findings may only be detected after the cohorts reach school-age, as observed in the MOMS trial.^(1)^

### Interpretation

We believe that this finding is crucial for the analysis of long-term neuroprotective techniques that are currently used for prenatal repair.^(15-22)^ The notion that the fetus is "just a small baby," and therefore, the same postnatal techniques can be applied has been proven wrong for several reasons.

The primary goal of postnatal repair is to promptly stop CSF leakage after birth to mitigate the risk of CNS infections. In contrast, prenatal intervention ensures that the fetus is "protected" from infection in the womb, and provides more time for the dural repair to naturally become watertight that culminates in the formation of a neoduramater.

In 97% of our cases, we observed reversal of hindbrain herniation, reinforcing the observation that biocellulose-based repair becomes watertight, even without sutures to repair the dura or secure the patch.

Another essential objective of prenatal repair is to preserve motor neurons in the placode. In an animal model, our group conducted tests on the effects of biocellulose placed above the placode without the need for sutures and compared it with conventional neurosurgical repair.^(23-27)^ The study comparing these approaches demonstrated inflammation, neuroanatomical disruption, and placode adhesion to the scar in the neurosurgical group. We posited that the differences were possibly attributable to the placement of penetrating sutures into the duramater that caused disruption and adhesion. Using the biocellulose patch, we observed no disruption of the neuroanatomy or adhesion, and a neoduramer was formed in all cases.

The concept of avoiding sutures to the dura has been analyzed in the literature. A recent study conducted in pediatric patients who underwent spinal surgery that utilized non-penetrating titanium clips to close the dura demonstrated a five-fold reduction in postoperative complications (CSF leaks and infections) as compared to dural repair with stitches.^(28,29)^ These authors believe that the holes caused by the passing of the suture in the dura increase the risk of CSF leakage, which can be avoided when using non-penetrating clips.

In the MOMS trial, the same neurosurgical technique was used for prenatal and postnatal repairs. At 12 months, 8% of patients in the prenatal group required surgery to untether the cord, whereas the rate was only 1% in the postnatal group.^(1)^ Although this difference was not statistically significant at that time (*p*=0.06), follow-up of the same cohort at school age revealed that the difference was statistically significant (p=0.03).^(8)^ The need for cord release was noted in 27% of the prenatal group *versus* 15% in the postnatal group (*p*=0.03). Tethering of the cord contributed to a 11% decrease in the ambulation rate in the prenatal group. At 30 months, 42% of the infants had independent ambulation whereas, at 6-7 years of age, the same cohort had a 29% ambulation rate.^(8)^

A potential reason for prenatal repair that resulted in more tethering may be related to the position of the medullary conus that, compared to that after birth (L3), is typically at a lower level (L4-5) before 25 weeks of gestation, which is the usual timepoint of prenatal surgery.^(5)^ This could lead to the medulla adhering to the spine at a lower level than when repair is performed postnatally, and makes the cord susceptible to progressive stretching as the child grows in height. Therefore, performing surgery between 26 and 28 weeks should be considered, and the belief that prenatal repair performed "the earlier, the better" may not hold true, because earlier repair may lead to more stretching as the conus typically ascends by one or more levels (from L4-5 to L3) gestationally.

Different groups have reported the need for cord untethering at various ages (Table 4);^(1,30-32)^ however, the TCS usually becomes more prevalent at or after school age. For example, Diehl et al. reported a 3% need for untethering at 12 months, but did not provide data at 30 months.^(30)^ On analyzing the data from the MOMS Group, we observed a threefold increase in the need for cord untethering from 12 months to that at 6-7 years (from 8% to 27%). The Texas Children’s Hospital (TCH) group reported that 18% of cases required cord untethering at 12 months, which was twice as much as that in the MOMS Group at the same timepoint.^(31)^ If the risk in the MOMS trial increased threefold at school-age, this would imply that TCS may occur in up to 50% of cases requiring cord untethering. Consequently, we believe that it is important to standardize repair techniques regardless of the approach, whether open surgery, laparotomy-assisted, or percutaneous fetoscopy, as the interventions may yield the same long-term benefits if the same biocellulose-based repair is used. In both cohorts, from 12 to 30 months and >30 months, the need for surgery to untether the cord was lower than that with all previously described techniques.

**Table 4:** Need for surgery to untether the cord in the largest series of prenatal repair

Surgery to untether cord	Present study n=136	Adzick (2011) MOMS prenatal^(1)^ n=79	Diehl (2021)^(30)^ and Graf (2016)^(32)^	Belfort (2020)^(31)^ n=49
12 months, %	3 (2/58)	8 (6/77)	3 (2/71)	18 (9/49)
30 months, %	5 (4/78)	No data	No data	–
6 years, %	–	27 (23/79)	–	–

The neurosurgical technique that differs the most from ours in terms of cord tethering is the one used in laparotomy-assisted fetoscopy. Notably, this technique began with an initial series of 10 cases that underwent non-standardized repair^(33)^ and subsequently evolved into two different standardized approaches: a single-layer repair performed in 32 patients and a three-layer repair in 18 cases.^(31)^ The single-layer group showed a 28% need for cord untethering at 12 months of age, whereas the three-layer group did not exhibit any cord tethering, although the sample was small as not all babies reached 12 months of age. When we summarized the combined data from the single- and three-layer groups, the total incidence of tethering reached 18% in the TCH cohort, whereas we observed a statistically significant difference of 3% in our study (Table 5).

**Table 5:** Comparison of the need for surgery to untether the cord between each one of three series and the present study

12 months	Tethered Yes	Tethered No	p value
Present study	2	58	reference
MOMS^(1)^	6	71	0.465
Germany^(30)^	2	69	1
TCH^(31)^	9	40	0.0216*

* Fisher’s *t*-test results were considered significant at p<0.05.

### Strengths and limitations

The neurosurgical technique we developed more than 14 years ago was the only technique that was tested and compared with the one used in the MOMS trial. All steps were tested in animal models and applied only in humans. Therefore, our study resulted in a truly translational approach from the bench to bedside. Additionally, we successfully replicated the myofascial flap described by the CHOP Group in 2015, which is currently used in open surgery.^(11)^

In animal studies, we compared biocellulose with various dermal matrices that were available at the time - that is, human acellular and collagen-based dermal matrices. We demonstrated that these grafts adhered to the placode, whereas biocellulose did not.^(26)^ Recently, other products have been investigated, such as the human umbilical cord matrix^(34)^ and synthetic biodegradable patch;^(35)^ however, their efficacy has not been proven.

Approval for biocellulose use as a drug substitute in humans was obtained in 2013 when the *Cirurgia Endoscópica para Correção Antenatal da Mielomeningocele* (CECAM) study was approved by the Brazilian National Ethics Committee. Recently, two prospective randomized trials have compared biocellulose with other commercially available dura mater patches as a dural substitute in craniotomies.^(36,37)^ These trials paved the way for the approval of the biocellulose as a dural substitute in humans by the FDA in January 2022 (SYNTHECEL Dura Repair™©DePuy-Synthes 2022, Raynham, MA, USA).

We report the outcomes of all cases, except one in our cohort; of the total 172 cases, only one case was lost to follow-up. This is very important when we compare our data to other studies that reported large numbers, but only when evaluating cases for which follow-up could be obtained.^(38)^ The selection bias in this retrospective study preclude any important conclusions, especially regarding "the earlier the gestational age of surgery, the lower the shunt rates."

Our series is the largest to report long-term outcomes from a single center. Our 78 cases 30-months or older can be compared with the 78 cases of prenatal surgery in the MOMS trial. In 61.8% (47/76) of the children, the only abnormality observed on the PEDI scale was related to motor function, with intact social and self-care functions. We would expect abnormal motor function in babies with OSB; therefore, we can consider these children normal except for their motor development.

Our primary concern was the prematurity rate, and we have been actively addressing this issue, although significant changes have not yet been achieved. Nevertheless, our mean gestational age at delivery, which is 33.2 weeks, is not significantly different from the 34.0 weeks reported in the MOMS trial and accepted by groups that use the open surgery approach for spina bifida repair.

A main limitation of this study is that TCS typically occurs as a late complication, often manifesting at school-age or later. In our cohort, 39 children were aged 6 years or older, which limits comparison to the school-age cohort of the MOMS trial. Another limitation stems from the fact that, because the repair technique is relatively new, it is possible that tethering may occur even later than reported by other groups. We will prospectively follow-up with our cohort, and any changes will be reported. Nevertheless, our series had the largest number of cases of biocellulose-based repair available in the literature, and significant differences from other series may potentially increase as more patients reach age 6. It is worth noting that biocellulose-based repair can be applied to both open surgery and laparotomy-assisted techniques; therefore, we believe that it is time for all groups to adopt the same technique.

## CONCLUSION

The need to untether the cord has compromised the long-term neuroprotective benefits of classical neurosurgical techniques for prenatal repair in infants, significantly reducing their ability to walk. Thus far, our cohort has shown the lowest rates of cord tethering at 12 and 30 months. We attributed this improvement to the use of biocellulose as a dural patch, the absence of sutures in the dura mater, and, potentially, the later gestational age at which we performed the surgery, typically between 26 and 27 weeks. We believe that, regardless of the approach used to reach the fetus, whether open or fetoscopic, a comparison of surgical techniques is warranted.
